# Outcomes of dual mobility arthroplasty in thumb basal joint arthritis: a clinical and radiographic study of one hundred and fifty prostheses with four-years follow-up

**DOI:** 10.1007/s00264-025-06639-5

**Published:** 2025-09-29

**Authors:** Giulia Frittella, Leopoldo Arioli, Matteo Guzzini

**Affiliations:** 1 Sapienza University of Rome, Rome, Italy; 2 Saint Camillus International University of Health and Medical Sciences, Rome, Italy

**Keywords:** Trapeziometacarpal osteoarthritis, Arthroplasty, Prosthesis, Dual mobility, Thumb

## Abstract

This study evaluated the outcomes of double mobility trapeziometacarpal prostheses for treating osteoarthritis (OA) of the trapeziometacarpal (TMC) joint. A prospective observational analysis was conducted on 150 implants with a maximum follow-up of four years, including a clinical and radiographic assessment and an evaluation of complications. The results indicate a prosthesis survival rate of 97.9% after the first two years post-surgery, calculated using the Kaplan-Meier method. Significant improvements were observed in pain reduction (mean VAS at 3 months post-surgery 2,9 and 1.5 after 6 months), hand grip strength (25.93 kg at 6 months post-surgery), and range of motion (Kapandji score from 8.8 to 9.2, comparing the preoperative mean with the mean after the first postoperative month). The complication rate was low, with only two cases of cup migration and one case of trapezium resorption. Patient satisfaction was high due to the rapid functional recovery and reduced invasiveness compared to traditional techniques. Double-mobility prostheses offer a highly effective treatment for stage II and III TMC OA according to the Eaton-Littler classification, with minimal need for revision surgeries.

## Introduction

Trapezio-metacarpal (TMC) joint osteoarthritis (OA) is the most frequent localization of osteoarthritis in the hand, especially in post-menopausal women [1–4]. The radiographic prevalence of this condition has been estimated to be 7.3% of the female adult population and 39% of the elderly female population [3]. Even though the severity of symptoms can vary among patients, this condition often leads to joint pain, weakness, and deformity with obvious consequences for the patient’s quality of life [2, 5].

Surgery is required when conservative treatments such as NSAIDs and steroid local injections fail, usually in patients with stage II and III according to Eaton Littler classification [6–8]. Surgical techniques are numerous, and trapeziectomy with or without biological arthroplasties has been considered the gold standard for many years [9–11]. However, recovery after this procedure is slow and often requires physical therapy to regain pinch strength. Moreover, the excision of the trapezium is an invasive procedure that alters the biomechanics of the thumb, usually leading to subsidence of the I metacarpal bone [11, 12].

Dual mobility trapeziometacarpal prostheses don’t require excision of the trapezium and offer a quick recovery from pain and weakness for patients with stage II and III OA [11]. From a biomechanical point of view, these implants restore thumb length thanks to an anatomical reduction of the joint [12–14]. Compared to the previous single-mobility prostheses, the new ones have drastically reduced the risk of dislocation and loosening [11].

The primary aim of this prospective study was to report and evaluate the clinical and radiological results of TOUCH^®^ and MAÏA™ double mobility prostheses with a maximum follow-up of four years. The secondary aim was to evaluate implant survival.

## Materials and methods

### Study design and participants

This is a retrospective review of prospectively collected data, corresponding to Level IV evidence. In this study, we analysed 138 patients with osteoarthritis of the trapeziometacarpal joint who underwent surgery between January 2020 and May 2024 in the same hospital, under the care of a level 4 hand surgeon [15].

All patients were operated on with a total joint replacement procedure with the use of a double mobility trapeziometacarpal prosthesis, followed by a clinical and radiological follow-up of up to four years. The surgical technique was the same for all patients, but two kinds of prostheses were used: TOUCH^®^ (Kerimedical; Geneva, Switzerland) and MAÏA™ (Lepine, Genay, France). Of the 138 patients, 16 underwent bilateral surgery: 12 received arthroplasty implants in both hands, and four were operated on with a different procedure on the contralateral hand (suspension arthroplasty, according to Altissimi [16]). Therefore, the total number of hands operated on with TM prosthesis implants was 150. The prostheses in bilaterally operated patients were always implanted with at least six-month intervals between procedures to allow for full recovery. The implantation of one prosthesis did not affect the recovery of the contralateral hand. Inclusion criteria were stage II or III thumb basal joint arthritis according to the Eaton Littler radiographic classification [17, 18]a reported history of significant pain at the base of the thumb, with functional limitation and reduced daily activities, and insufficient response to at least three months of conservative treatment, such as oral NSAID therapy and local corticosteroid injections.

Exclusion criteria were stage IV osteoarthritis, particularly if the scaphotrapezial (ST) joint surface was affected, previous trapezium fracture or dysplasia, and insufficient trapezium bone height (< 8 mm) measured with x-ray 1:1 in three projections.

All patients were extensively informed about the surgical procedure, and valid consent was collected for the surgery and participation in the study.

The study was in line with the Ethical Standards of the 1975 Declaration of Helsinki, as revised in 2008.

### Preoperative data

The preoperative clinical assessment was performed using a visual analog scale (VAS) for pain measurement and using the Quick Disabilities of the Arm, Shoulder, and Hand Questionnaire (Quick DASH) for evaluation of upper limb function. Moreover, the opposition of the thumb was assessed using Kapandji’s score, and ROM of the TMC joint was measured (radial abduction) along with hand grip, tip pinch, and key pinch strength (Jamar^®^ dynamometer, White Plains, CT, USA).

All patients had preoperative X-ray exams, in the radiographic evaluation, osteoarthritis of the TMC joint was staged using the Eaton Littler classification [17–19]if present, OA of the STT joint was recorded. The M1/M2 ratio [14] was calculated to assess the preoperative length of the thumb and compare it to the post-operative one.

### Postoperative data

The same clinical and radiological evaluations were used in the post-operative assessment. The follow-up consisted of appointments at one, three, six and 12 months during the first year, followed by annual appointments in the subsequent years. The presence of any complication, either clinical or radiographic, was noted.

### Operative technique and prosthesis

The procedure was performed under brachial plexus block and a tourniquet was applied. A first-generation cephalosporin was given intravenously as per preoperative prophylaxis guidelines.

In this study, two types of TM prostheses were implanted, both press-fit, modular, and dual mobility. The Touch^®^ prosthesis consists of a conical or hemispherical cup in two sizes, a modular straight or 15° off-set neck in three lengths, and an anatomical metacarpal stem with a trilobed section of six dimensions. The cup and the stem are made of stainless steel coated with a dual coating of porous titanium and hydroxyapatite, while the insert is made of polyethylene.

The Maïa™ prosthesis consists exclusively of a spherical cup in two sizes, a modular 15° off-set neck of three lengths, and a stem of four dimensions. The cup and the stem are made of titanium alloy coated with porous titanium and hydroxyapatite, while the insert is made of polyethylene. For patients allergic to nickel, titanium necks are available.

A dorsolateral longitudinal incision of approximately 3 cm was made to expose the TM joint between the extensor pollicis brevis (EPB) and the extensor pollicis longus (EPL). The radial nerve superficial branches and the radial artery dorsal branch were isolated and retracted. The joint was exposed with a longitudinal capsule incision. If present, osteophytes on the base of the first metacarpal bone were removed and a 5 mm osteotomy was performed. The medullary canal was then prepared with rasps of increasing size until the correct size was found. At this point, the corresponding trial stem was placed.

All osteophytes and horns were removed after exposing the trapezium with a retractor. A centering guide and intraoperative fluoroscopy were used to position a K-wire as a guide for the cup reamers. The K-wire should be perpendicular to the scaphotrapezial joint surface and centered in the trapezium. After using reamers in different sizes, a trial cup was positioned in the trapezium. Before implanting the definitive components, stability and range-of-motion tests were performed. The capsule joint was then sutured, and after obtaining haemostasis, the skin was sutured with absorbable stitches.

### Postoperative care

The postoperative care consisted of a wound dressing scheduled after the first and the second-week post-op. After 14 days patients were encouraged to resume a light activity with the operated hand. Physical therapy was prescribed based on clinical evaluation of the range of motion of the thumb joint, trophism of the thenar eminence, and functional recovery of the hand. If prescribed, physical therapy was scheduled with a frequency of three to five appointments per week.

### Statistical analysis

We considered the prostheses (*n* = 150) as the statistical unit assuming that the surgeries were independent, even in patients who underwent bilateral operations. In these cases, the surgeries were performed at least six months apart. One surgery did not affect the recovery of the other. Clinical and radiographic data collected during follow-up were compared with preoperative data. Descriptive statistics were calculated as means, standard deviations (SD), and confidence interval limits. Two software programs, SPSS and Jamovi, were used for data analysis. The prosthesis survival curve was calculated using the Kaplan-Meier method. Failure was defined as prosthesis removal and conversion to Trapeziectomy.

## Results

### Clinical outcomes

#### Patients

The number of patients that satisfied all inclusion criteria was 138. 12 of them were operated bilaterally, therefore 150 prostheses were implanted. The patient demographic is shown in Table n 1. 21 patients were affected by other conditions that required surgical procedures carried out during the same operation, they are shown in Table n 2.

119 of the 150 implanted prostheses were TOUCH^®^ while 31 were Maïa.

### Clinical results

Both Touch^®^ and Maïa™ prostheses resulted in significant improvements in pain, hand function, and strength over time.

For the Touch^®^ group (*n* = 119), the average VAS score at rest decreased from 7.3 preoperatively to 1.3 at 12 months. The QuickDASH score improved from 37.5 to 12.1 at 12 months, and hand grip strength increased from 22.7 kg to 27.9 kg at 12 months, reaching 34.9 kg at 48 months. The average Kapandji score improved from 8.8 preoperatively to 9.7 at 12 months, with most patients reaching 10 at later follow-ups. Key and pulp pinch also improved progressively across follow-ups.

For the Maïa™ group (*n* = 31), the pain reduction trend was similar, with average VAS dropping to 1.5 at 12 months, and hand function scores improving in parallel. Mean DASH at 12 months was 11.0, and grip strength averaged 31.0 kg. The Kapandji score reached 10 in the majority of patients by one year. Although the sample size was smaller, no clinically relevant differences were noted compared to the Touch group.

After four years of follow-up, most patients had an excellent satisfaction rate. 98% of patients who reached final follow-up declared that they would choose to have the same surgery on the contralateral hand if needed.

Clinical outcomes are presented in Table 4.

### Radiological findings

The mean Eaton Littler’s stage was 2,81+/-0,3 in the preoperative radiological assessment. Regarding the M1/M2 ratio, our preoperative data showed a mean of 0,73+/0,06 while the post-operative values were calculated to be 0,76+/-0,02. These values remained stable over time for most patients, proving the good integration of the prostheses with no impaction or migration of the components. Similar findings were observed both in Touch and in Maia implants.

We recorded four cases of radiolucent zones around the stem (3 of them had stage 1 according to Lussiez’s classification [11] and 1 had stage 2). None of them had any clinical discomfort related to this radiological finding.

### Complications and implant survival

Kaplan-Meier survival analysis was conducted separately for each prosthesis type. Survival estimates and 95% confidence intervals (CI) are presented in Table 3. No implant failures were observed in the Touch^®^ group during the first 12 months, resulting in an estimated survival rate of 100% for that period. Both recorded failures (one per group) occurred after 12 months postoperatively. No Maïa™ prosthesis has yet reached 48 months of follow-up, limiting long-term survival estimation for this group.

**Table 1:** Demographic characteristics of patients undergoing trapeziometacarpal arthroplasty

Number of patients	138
Age, mean ± SD (range), years	64.36 ± 7.6 (44–84)
Gender	
- Males	28 (20.2%)
- Females	110 (79.8%)

**Table 2:** Additional hand conditions treated during the same surgical procedure

Condition	Incidence
Just prostheses implant	129 (86.0%)
+ Dupuytren’s disease	2 (1.3%)
+ De Quervain’s syndrome	1 (0.7%)
+ Trigger Finger	7 (4.7%)
+Carpal Tunnel Syndrome (CTS)	10 (6.7%)
+CTS + Trigger Finger	1 (0.7%)

**Table 3:** Kaplan-Meier survival estimates of the prostheses at 12, 24, and 48 months

Prosthesis	Month	Estimated Survival	95% CI	Failures
Touch^®^	12	100%	—	0
Touch^®^	24	98.4%	95.4% – 100%	1
Touch^®^	48	98.4%	95.4% – 100%	1
Maïa™	12	96.4%	89.6% – 100%	1
Maïa™	24	96.4%	89.6% – 100%	1

**Table 4:** Clinical outcomes (VAS, DASH, hand strength, Kapandji score) recorded at baseline and follow-up visits

Test	Pre-op	1 mo	3 mo	6 mo	12 mo	24 mo	36 mo	48 mo
VAS	7.3 ± 1.43	4.5 ± 1.33	2.9 ± 0.75	1.5 ± 1.58	1.3 ± 1.86	1.12 ± 1.25	0.55 ± 1.14	0.27 ± 1.15
DASH	37.5 ± 2.4	25.2 ± 12.9	19.3 ± 11.7	15.42 ± 10.01	12.07 ± 7.8	12.15 ± 8.46	4.80 ± 0.6	2.76 ± 0.88
Hand Grip (kg)	22.7 ± 11.2	16.23 ± 8.5	16.56 ± 7.7	25.93 ± 10.6	27.86 ± 10.9	32.71 ± 11.1	34.2 ± 10.7	34.9 ± 11.3
Tip Pinch (kg)	4.39 ± 5.7	2.6 ± 1.8	2.7 ± 1.6	3.2 ± 1.6	4.01 ± 2.3	5.5 ± 2.66	5.8 ± 1.95	5.8 ± 2.3
Key Pinch (kg)	4.39 ± 2.81	3.64 ± 1.9	4.01 ± 1.7	4.35 ± 1.2	5.7 ± 2.5	6.54 ± 2.09	6.71 ± 1.87	6.82 ± 2.1
Kapandji	8.8 ± 1.01	9.2 ± 1.01	9.6 ± 0.5	9.5 ± 0.7	9.7 ± 0.5	9.8 ± 0.35	9.8 ± 0.6	9.9 ± 0.7

Three major complications led to revision. One patient had a cup tilting (Touch^®^), and one had a proximal cup migration (Maïa™) due to trapezium resorption. Both were treated surgically by removing the prosthesis’ components and performing trapeziectomy with suspension arthroplasty. In another case, migration of the Maïa™ implant occurred secondary to a trapezium fracture, which was treated conservatively based on the patient’s preference. A splint was applied for four weeks, and NSAIDs were administered for pain management. Thumb opposition was slightly reduced compared to the pre-injury status (Kapandji score decreased from 9 to 8); however, pain management was effective, and the overall outcome was acceptable given the conservative treatment approach.

Minor complications included ten cases of De Quervain’s syndrome. While four patients experienced mild symptoms that resolved spontaneously without the need for medical treatment, five other patients were treated with steroid injections and one with surgery.

At 12 months, 66 Touch^®^ and 28 Maïa™ prostheses were actively followed. At 24 months, 64 and 28, respectively, remained in follow-up. At the time of analysis, 50 Touch^®^ implants had reached 36 months of follow-up, and 40 Touch^®^ implants had completed the 48-month follow-up. No Maïa™ prostheses had yet reached 36 or 48 months. This progressive reduction in follow-up numbers is due to the timing of surgeries, as many implants were placed in the latter years of the study period and had not yet reached the longer-term timepoints.

## Discussion

To our knowledge, this is currently the largest Italian clinical series of dual mobility trapeziometacarpal prostheses, with up to four years of follow-up. What makes this study particularly relevant is the inclusion of a comprehensive and separate evaluation of two different implant systems (Touch^®^ and Maïa™), with detailed analysis of clinical and radiographic outcomes, as well as Kaplan-Meier survival curves with 95% confidence intervals. This approach, rarely seen in previous studies, offers valuable insights into implant performance and mid-term patient satisfaction. Considering the growing popularity of total joint arthroplasty for thumb basal joint osteoarthritis in Europe, our results contribute significantly to the available evidence and may support future decision-making in surgical practice.

This study demonstrated that dual-mobility trapeziometacarpal prostheses are an excellent option for treating thumb osteoarthritis after a four year follow-up with high patient satisfaction.

Trapeziectomy has always been considered the gold-standard treatment for osteoarthritis of the thumb and still is today. Even though a multitude of biological arthroplasty techniques have been published over the years, no study has determined if one technique is superior to the others [7, 9, 20, 21]. 

Trapeziectomy with arthroplasty is a good and reliable procedure characterized by a low rate of complications and revision surgeries, ensuring pain reduction and recovery of function. However, it is not a technique without weaknesses. The procedure involves the excision of the trapezium, thereby altering the entire biomechanics of the thumb. Subsidence of the first metacarpal (MC) is a very frequent consequence, with impingement on the scaphoid and a reduction in pinching strength [12, 22, 23]. 

Even arthroplasties are not free from complications, such as component wear, dislocations, aseptic loosening of components, and trapezium fractures [7, 11, 12, 24, 25]. However, many published studies currently suffer from strong biases, represented by small studies, short-term follow-ups, and the use of non-standardized surgical techniques and trapeziometacarpal prostheses that have different characteristics.

The most recent literature shows implant survival of 95% even after ten years from surgery [9, 11, 26] and the results of our study are in line with the current literature, albeit with a follow-up of only four years: 99.1% survival after one year from surgery and 97.9% after two years.

Recent literature also shows the superiority of trapeziometacarpal prostheses compared to previous techniques in restoring the height of the first ray, with better recovery of range of motion (ROM) and grip strength [12, 13]. Our patients showed an excellent recovery of strength as early as three months postoperatively (Hand grip 16.56 kg at three months postoperatively and 25.93 kg six months after surgery). Recovery of opposition occurs as early as the first month postoperatively, with an average Kapandji test score of 9.2 just one month after surgery (preoperative 8.8).

Regarding pain relief, significant improvement occurs as early as the first month (mean VAS reported 4.5), and six months after surgery the average reported pain level stabilizes at 1.5 out of 10. According to Ulrich [12]the use of trapeziometacarpal prostheses, given the rapid functional recovery, quick return to daily activities, and substantial disappearance of pain within the first three months postoperatively, represents an indication for treatment, especially in patients with moderate functional demands but with a work or hobby priority that requires a quick return to daily activities.

Regarding our radiological assessment, the mean M1/M2 ratio increased from 0.73 ± 0.06 preoperatively to 0.76 ± 0.02 postoperatively, in line with current literature and studies by Ledoux published in 2017^25,30^, which highlighted the importance of restoring the physiological height of the first ray to reproduce the normal biomechanics of the thumb.

However, our study has some limitations. Firstly, our four year follow-up period is currently considered a short period to evaluate the effectiveness and complications associated with trapeziometacarpal prostheses, not reaching the minimum threshold of five years necessary for medium-term follow-up, with several studies in the literature now having long-term follow-up exceeding ten years. This point is particularly important when considering the survival of implanted prostheses. Nevertheless, a prosthesis survival rate of 97.9% at four years should be considered an excellent result, as numerous studies on the survival of various TM prostheses report a higher failure rate as early as the first year of implantation, suggesting that higher failure rates may be related to the surgeon’s learning curve and implant characteristics. Additionally, our study does not include a comparison cohort, potentially composed of patients who underwent simple trapeziectomy or biological arthroplasty which could have provided useful information regarding the actual differences between the two types of treatment. Therefore, randomized controlled clinical trials are needed to confirm the performance of these new prostheses.

## Conclusions

Our study suggests that trapeziometacarpal dual-mobility prostheses currently represent an excellent treatment option for stage II and III OA, where the scapho-trapezio-trapezoid joint is not involved.

TMC prostheses allow for the restoration of the physiological height of the first ray and a return to normal biomechanics. This helps reduce the classic “Z” deformity and avoids the first ray subsidence with scaphoid-metacarpal impingement, which is common in alternative techniques that involve trapeziectomy. Additionally, preserving the trapezium reduces the invasiveness of the surgery and maintains the option of performing a rescue trapeziectomy should complications arise.

Trapeziectomy with or without biological arthroplasty should continue to be considered the first choice of treatment for patients with stage IV OA or as a rescue technique.

™ | 24 | 96.4% | 89.6% – 100% | 28 | 1 |

## Data Availability

No datasets were generated or analysed during the current study.
